# The risk-takers and -avoiders: germination sensitivity to water stress in an arid zone with unpredictable rainfall

**DOI:** 10.1093/aobpla/plz066

**Published:** 2019-10-10

**Authors:** Corrine Duncan, Nick L Schultz, Megan K Good, Wolfgang Lewandrowski, Simon Cook

**Affiliations:** 1 School of Health and Life Sciences, Federation University, Mt Helen, VIC, Australia; 2 BioSciences, University of Melbourne, Parkville, VIC, Australia; 3 Kings Park Science, Department of Biodiversity, Conservation and Attractions, Kings Park, WA, Australia; 4 School of Biological Sciences, The University of Western Australia, Crawley, WA, Australia

**Keywords:** Ψ _b_50, *t*_50_, bet-hedging, cardinal temperatures, hydrotime, seed mass, seed physiology, water potential

## Abstract

Water availability is a critical driver of population dynamics in arid zones, and plant recruitment is typically episodic in response to rainfall. Understanding species’ germination thresholds is key for conservation and restoration initiatives. Thus, we investigated the role of water availability in the germination traits of keystone species in an arid ecosystem with stochastic rainfall. We measured seed germination responses of five arid species, along gradients of temperature and water potential under controlled laboratory conditions. We then identified the cardinal temperatures and base water potentials for seed germination, and applied the hydrotime model to assess germination responses to water stress. Optimum temperatures for germination ranged from 15 to 31 °C under saturated conditions (0 MPa), and three species had low minimum temperatures for germination (<3 °C). A small proportion of seeds of all species germinated under dry conditions (Ψ ≤ −1 MPa), although base water potential for germination (Ψ _b_50) ranged from −0.61 to −0.79 MPa. Species adhered to one of two germination traits: (i) the risk-takers which require less moisture availability for germination, and which can germinate over a wider range of temperatures irrespective of water availability (*Casuarina pauper* and *Maireana pyramidata*), and (ii) the risk-avoiders which have greater moisture requirements, a preference for cold climate germination, and narrower temperature ranges for germination when water availability is low (*Atriplex rhagodioides*, *Maireana sedifolia* and *Hakea leucoptera*). High seed longevity under physiological stress in *H. leucoptera*, combined with a risk-avoiding strategy, allows bet-hedging. The hydrotime model predicted lower base water potentials for germination than observed by the data, further supporting our assertion that these species have particular adaptations to avoid germination during drought. This study provides insights into the complex physiological responses of seeds to environmental stress, and relates seed germination traits to community dynamics and restoration in arid zones.

## Teaser

In an arid ecosystem with unpredictable rainfall, we investigated the germination traits of keystone trees and shrubs. Species adhered to one of two germination strategies: (i) the risk-takers which require less moisture availability for germination and have wide temperature ranges for germination (*Casuarina pauper* and *Maireana pyramidata*), and (ii) the risk-avoiders that have greater moisture requirements, a preference for cold climate germination, and narrower temperature ranges for germination (*Atriplex rhagodioides*, *Maireana sedifolia* and *Hakea leucoptera*). We suggest that these species have particular adaptations to avoid germination during drought, and this is supported by low base water potentials for germination.

## Introduction

Plant recruitment in the arid zone is infrequent and episodic due to water limitation (Schwinning and Sala 2004; Wiegand *et al.* 2004). Understanding the determinants of recruitment losses is crucial for the restoration of ecosystems affected by changes to land use and climate (Commander *et al.* 2019). Seed germination is a critical life transition stage for arid plants and is largely controlled by water availability (Adams 1999); hence, moisture conditions must overlap with species’ germination traits (Clauss and Venable 2000; Köchy and Tielbörger 2007). Some arid plant species adopt a risk-taking strategy, and produce seeds with low moisture thresholds that germinate in response to small rainfall events (Ramírez-Tobías *et al.* 2014), while others adopt a risk-averse strategy so germination occurs only in wet soils (Sfairi *et al.* 2012; Mollard and Naeth 2015; Merino-Martín *et al.* 2017). As such, small rainfall events may only affect species with a risk-taking strategy and fast response times, while larger rainfall events are required to stimulate germination and support the establishment of risk-avoiders—generally higher vascular plants and slow-growing species, including tree species (Noy-Meir 1973; Schwinning and Sala 2004). We assume that seed germination in arid zones occurs mostly in response to large rainfall events (Gutterman 1994), yet species responses to different-sized rainfall events are rarely quantified (but see Meyer and Allen 2009), even though they ultimately determine community dynamics in arid zones (Reynolds *et al.* 2004).

The emergence of germinated seed appears to be the major recruitment bottleneck for many arid species (Pyke 1990; Chambers 2000; James *et al.* 2011). Moisture availability in arid zones is driven by rainfall, and is a crucial determinant of germination, seedling growth and the distribution patterns of species (Gutterman 1993). Generally both germination rate and germination proportion decrease progressively with decreasing soil water potential (Bradford 1990). As ambient temperatures increase, so does evaporation and evapotranspiration (Feng *et al.* 2014), and the temporal period of moisture availability is decreased (Wang *et al.* 2012). Seeds that germinate when moisture is available for short periods face the risk of emerging during conditions unfavourable for seedling establishment (Gremer and Venable 2014). Some arid species show particular adaptive germination mechanisms to restrict germination to wetter periods (Zeng *et al.* 2010) or to avoid germination during summer temperatures (Sánchez *et al.* 2014). However, physiological thresholds for germination in a range of native species from within the same climatic origin are rarely assessed (Köchy and Tielbörger 2007; Hu *et al.* 2015; Flores *et al.* 2017) as most studies focus on temperature dynamics only (e.g. Lai *et al.* 2016). Defining thresholds for germination has proven useful for crop and weed emergence models (Forcella *et al.* 2000; Dürr *et al.* 2001; Gardarin *et al.* 2012); however, it has not been widely applied to understand recruitment and survival in native species from unpredictable environments. When species response thresholds are empirically tested and quantified, there is potential to predict community structure under climate change and specific management techniques (James *et al.* 2013).

Physical traits of seeds, and adult plant traits, are often used as a proxy for germination strategy (e.g. Moles and Westoby 2004; Hoyle *et al.* 2015). Large seeds may have an increased chance of seedling survival and establishment under dry conditions (Leishman and Westoby 1994; Moles and Westoby 2004; Daws *et al.* 2008), although small seeds tend to germinate faster than heavy seeds (Vivrette 1995), which is considered an important advantage for arid species (Chesson *et al.* 2004). The importance of the seed-size water-potential relationship varies among biomes (Metzner *et al.* 2017) and is difficult to predict under small moisture gradients (Tielbörger and Petru 2008; García-Baquero *et al.* 2015). Furthermore, annual plants tend to show a negative relationship between seed mass and base water potential for germination, whereas perennials display a negative relationship between base temperature for germination and seed mass (Arène *et al.* 2017). Hence, the link between seed size and hydrothermal thresholds to germination remains unclear and warrants further testing.

Germination is also linked to a species’ ecological niche (Arène *et al.* 2017), and the literature suggests differences in opinion of the influence of the environment in prescribing germination niches (Losos 2008; Vandelook *et al.* 2008; Fang *et al.* 2017). Certainly, plant taxa that are often associated with dry or saline environments, such as *Atriplex*, have greater tolerance ranges to water stress than other taxa (Deng *et al.* 2014; Shaygan *et al.* 2017). The interplay between adult plant traits and climate has been widely studied (Pérez-Harguindeguy *et al.* 2013; Sack *et al.* 2013), but we lack understanding of the link between germination thresholds, climate and seed traits (with the exception of seed size; Moles *et al.* 2007; Arène *et al.* 2017). Strong relationships exist between minimum temperatures for germination and the climatic conditions of biomes that species inhabit (Rosbakh and Poschlod 2015), and between seed size and base water potentials for germination (Daws *et al.* 2008; Arène *et al.* 2017) but, to our knowledge, no studies have explored how the unpredictability of rainfall may influence the seed traits and germination thresholds of native species.

Hydrotime models quantify the effects of water potential on seed germination and provide a useful tool to assess germination sensitivity relative to environmental conditions (Bradford 2002). These models are based on the linear increase in germination rates from base (*T*_b_), through optimum (*T*_opt_) temperatures for germination, and the steady decline in germination rate as conditions dry (Gummerson 1986; Bradford 1990, 2005). Although departures of actual seed germination from the hydrotime model are frequently reported at suboptimal and supra-optimal temperatures (Kebreab and Murdoch 1999; Grundy *et al.* 2000), it can be a useful tool in defining seed responses to microclimate conditions, and the germination niche of seeds (Bloomberg *et al.* 2009; Watt *et al.* 2011). Few studies have defined the physiological thresholds for seed germination from wild species in arid zones (Arnold *et al.* 2014b; Hu *et al.* 2015; Lewandrowski *et al.* 2016; Frischie *et al.* 2019); hence, our understanding of the role of water availability in the germination strategy of native species in the arid zone is limited.

Germination triggered by small rainfall events is risky, particularly when the chance of follow-up rainfall is low; hence, we predict high moisture thresholds for germination as the dominant strategy. We expect a higher proportion of seeds to germinate in cool temperatures, when moisture from rainfall events remains in soil for longer due to reduced evaporative water loss. We also expect a positive correlation between seed mass and base water potential for germination. This study provides insight into the complex germination behaviour of non-dormant, arid seeds, and relates seed germination traits to community dynamics in arid zones.

## Methods

### Seed collection and location

Seeds were collected from arid, south-west New South Wales (33°22′05″S, 142°13′36″S), from remnant populations targeted for restoration. Vegetation at the study site is classified as Belah-Rosewood Woodland and Belah-Pearl Bluebush Woodland (Sluiter and Sluiter 2015). The tree species that dominate these woodland communities are *Casuarina pauper* (Casuarinaceae), with smaller patches of *Alectryon oleifolius* ssp. *canescens* (Sapindaceae). Other tree species that appear as scattered individuals across the landscape include *Myoporum platycarpum* ssp. *platycarpum*, *Geijera parviflora* (Scrophulariaceae), and *Hakea* species including *H. leucoptera* ssp. *leucoptera* and *H. tephrosperma* (Proteaceae). The understory is dominated by *Maireana sedifolia*. Chenopod shrubs are common, but less prevalent, and include *M. pyramidata* and *Atriplex* species, such as *A. rhagodioides*.

**Figure 1.**
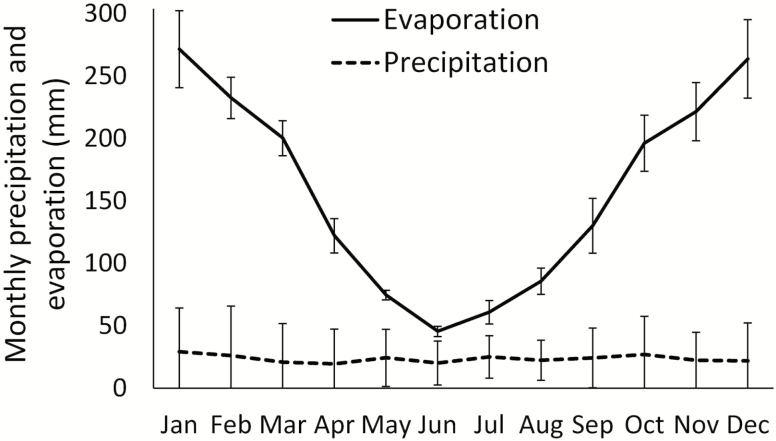
Mean monthly precipitation, from 1956 to 2016 (BOM 2018) and mean monthly evaporation, from 2013 to 2018 (Environdata 2018) at the study site. Error bars represent standard deviation.

The five keystone arid species included in this study are found in the remnant vegetation of the region—two trees (*C. pauper* and *H. leucoptera*), and three shrubs (*A. rhagodioides*, *M. sedifolia* and *M. pyramidata*). Mean monthly rainfall at the study site is 24 mm (BOM 2018) and average annual rainfall can often fall below 200 mm for consecutive years. Temperatures range from 2 to 47 °C with cooler mean daily temperatures from May to August. Evaporation is higher than rainfall across all months (Fig. 1) and, unlike most arid zones across the globe, there is no distinct wet season.

### Seed collection and processing

Fresh seeds were collected for testing from within a 100-km radius of the centre of the study area. Seeds were either personally collected (C.D.), donated from Ecotypic Pty Ltd and Tronox Mining, or purchased from Ogyris Pty Ltd. All species included in this study were non-dormant, except for *A. rhagodioides*, which has physiological dormancy that was alleviated through after-ripening for 18 months prior to the experiment (Duncan *et al.* 2019). Seeds were manually cleaned and all seed-covering structures, such as bracts, were removed. Seed mass was determined using the mean of three replicates of 100 seeds each, with results then adjusted to account for seed fill (determined by longitudinal cut tests) and divided by 100 to represent weight (g) per seed.

### Germination under water and temperature regimes

Seeds were incubated under eight temperatures from 5 to 40 °C (at 5 °C increments), and nine water potential treatments from saturated to wilting point (0, −0.01, −0.15, −0.25, −0.35, −0.5, −0.75, −1.0 and −1.5 MPa), applied through different polyethylene glycol (PEG) solutions (PEG-8000, Sigma Aldridge, Sydney, NSW, Australia). As such, there were 72 treatment combinations in total. The PEG solutions were prepared in water and calculated according to Michel (1983). Three replicates of 30 seeds per species were used for each water potential treatment, except for two species. To overcome low seed viability (~35 %) of *C. pauper*, the number of seeds per replicate for this species was increased to 40. For *H. leucoptera* the number of seeds per replicate was reduced to 25 due to low seed availability. Seeds were incubated in a 90-mm Petri dish on filter paper moistened with 5 mL of the relevant PEG solution and sealed tightly with cling film before, and during, incubation. To prevent microbial outbreak and ensure constant hydration during germination tests, seeds were transferred to sterilized Petri dishes weekly, on new filter papers moistened with the same appropriate PEG solutions. Seeds were incubated under alternating 12-h light/dark schedule incubators (Lindner and May, Model: LMRIL 396, Windsor, Australia), provided by 2 × 36-Watt fluorescent globes. Prior to germination treatments, seeds of all species were surface-sterilized by soaking in 1 % sodium hypochlorite for 1 min, then rinsed for 40 s with double distilled water. Seed germination (when the radicle emerged to at least half of seed size) was recorded every second day. Germination was scored for 30 days, or until germination ceased for four consecutive readings across all treatments. Incubator temperature was monitored every 2 days and the experiment was repeated when the temperature fluctuated 2 °C or more for two consecutive readings.

Seed viability was assessed by dissecting seeds after staining in a 1 % solution of 2,3,5-triphenyl tetrazolium chloride (TZ), except for *H. leucoptera* which was germinated on filter paper at warm diurnal temperatures due to consistently poor TZ stain results and a germination response of 100 %. Embryos that completely absorbed the TZ stain were scored as viable, and embryos that only partially absorbed the TZ stain were recorded as non-viable seeds. Seed viability was assessed within 2 days prior to the experiment and final viability-adjusted germination (VAG, herein referred to as germination proportion) was calculated using the following equation (Sweedman and Merritt 2006):

$$\begin{array}{l} VAG=\frac{\text{Final germination}(\%)}{\text{Mean viability}(\%)}\times 100 \end{array}$$

The effects of temperature and water stress on embryo health were assessed by performing further seed viability tests at the end of the experiment, by dissecting and staining seeds using the TZ methods mentioned above.

### Calculation of cardinal temperatures

The time to 50 % germination (*t*_50_) was determined by fitting a sigmoid curve to the mean values of germination proportion over time for each species, at each water potential. The sigmoid model describes the cumulative germination proportion (*G*) over time (*t*) and is described by:

$$\begin{array}{l} G=\frac{G_{max}}{1+e^{-a(t-b)}} \end{array}$$

where *G*_max_ is the maximum germination percentage, *t* is the time required for specific germination fractions, and *a* and *b* are constants. Estimates of cardinal temperatures were calculated using germination rate (GR = 1/*t*) at 0 MPa, plotted against temperature. We used the segmented package in R (Muggeo 2008; R Core Team 2018), which used iterations to fit a two-piece segmented linear model to the data for germination rate over time. From the two linear regressions in each segmented model, the intercepts with the temperature axis provided estimates for *T*_b_ and *T*_c_, respectively, and *T*_opt_ is the temperature at which the two linear regressions intercept (e.g. Frischie *et al.* 2019). Temperatures from the base (*T*_b_) to optimum (*T*_opt_) were species suboptimal temperatures for germination. Temperatures from *T*_opt_ to ceiling (*T*_c_), or maximum temperatures, where germination rate decreases, were species supra-optimal temperatures for germination. In species with rapid germination rates (*M. sedifolia* and *M. pyramidata*), the supra-optimal temperature range was exceptionally and atypically small, and the experiment did not capture the decrease in germination rate above *T*_opt_, such that the germination rate at the next 5 °C increment was 0. In these cases, the first temperature after *T*_opt_ provided an estimate of *T*_c_, though we acknowledge the actual value of *T*_c_ will be in the small temperature range between *T*_opt_ and our estimate of *T*_c_.

### Modelling base water potentials for germination

To determine the base water potential for 50 % germination (Ψ _b_50), we created a linear model to describe germination proportion at different water potentials using the experimental data. From this relationship, we solved for the water potential at 50 % germination. We then compared Ψ _b_50 at each temperature to parameters generated by the hydrotime model which uses the following probit regression analysis (Dahal and Bradford 1990):

$$\begin{array}{l} Probit(g)=\;[\Psi (\Psi_{H}/t_{g})\Psi_{b}(50)]\;/\sigma \Psi_{b} \end{array}$$

where Ψ is the seed water potential, θ _H_ (MPa h^−1^) is the hydrotime constant, *t*_g_ is the germination time (h) of the corresponding germination fraction, Ψ _b_(50) is the base or threshold water potential required to achieve 50 % germination of the seedlot and σΨ _b_ is the standard deviation. Calculation of hydrotime parameters was performed using the population-based threshold model spreadsheet, developed by UC Davis Department of Plant Science. Correlations between seed weight and Ψ _b_50 were assessed using a linear model in R (R Core Team 2018).

## Results

### Germination proportion effected by temperature and water potential

**Figure 2.**
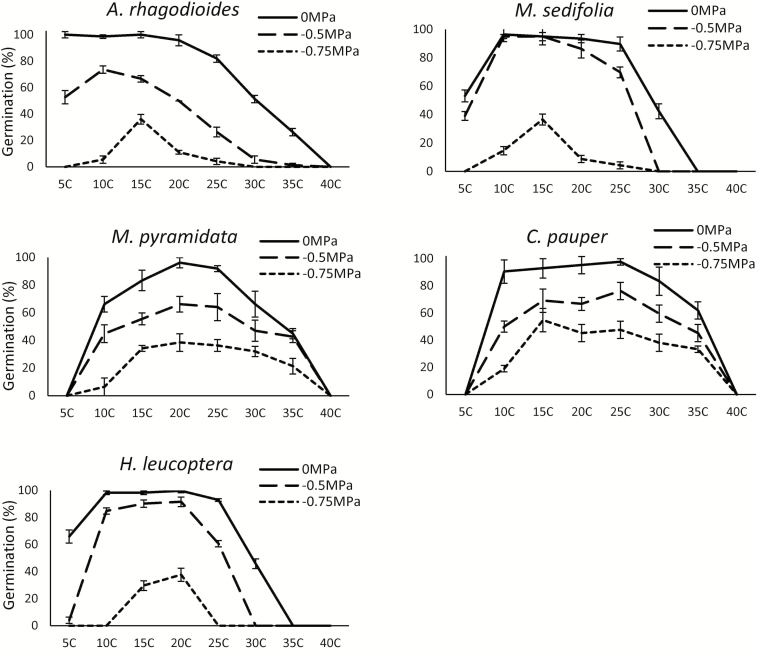
Final seed germination (mean ± standard error) of *A. rhagodioides*, *M. sedifolia*, *M. pyramidata*, *C. pauper* and *H. leucoptera* incubated under the combination of three water potentials (0, −0.5 and −0.75 MPa) and eight temperature treatments (5–40 °C).

Under saturated conditions (0 MPa), all species germinated within the 30-day experiment when temperatures were 10–30 °C (Fig. 2). No germination occurred at 40 °C for any species, while *H. leucoptera* and *M. sedifolia* also showed no germination at 35 °C. The only two species that failed to germinate at the lowest temperature tested (5 °C) were *C. pauper* and *M. pyramidata*. These were also the only two species to germinate to 50 % at 35 °C. While temperature envelopes for germination were wide under saturated conditions (0 MPa), they were much narrower in dry conditions, particularly for *A. rhagodioides*, *M. sedifolia* and *H. leucoptera* (Fig. 2). At water potentials ≤−0.75 MPa, no germination occurred at temperatures >30 °C for *A. rhagodioides* and *M. sedifolia*, and at temperatures ≥25 °C for *H. leucoptera*.

**Figure 3.**
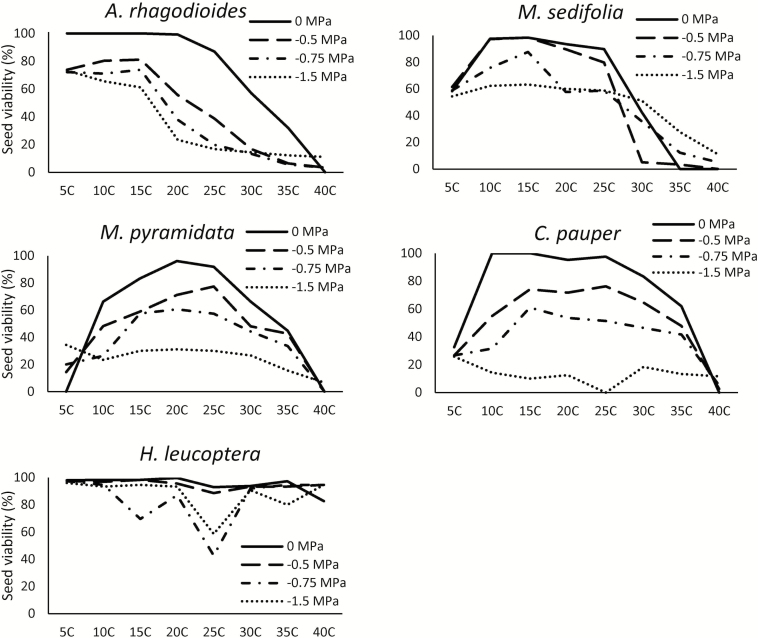
Seed viability of five species after 30 days of treatment at four different water potentials (0, −0.5, −0.75 and −1.5 MPa) and eight temperature treatments.

Three types of responses were observed regarding the viability of seeds exposed to temperature and water stress. Seeds were either killed by high temperatures of >35 °C (observed in *A. rhagodioides* and *M. sedifolia*), killed by negative water potentials of <−0.75 MPa (*C. pauper* and *M. pyramidata*) or, for *H. leucoptera*, remained viable after all temperature and water potential treatments (Fig. 3). *Hakea leucoptera* exhibited great resilience to temperature and water stress, because nearly all seeds remained viable after the 30-day germination experiment at lowest water potential (−1.5 MPa), and at hottest (40 °C) and coldest (5 °C) temperatures tested.

### Germination speed is affected by temperature and water potential

**Figure 4.**
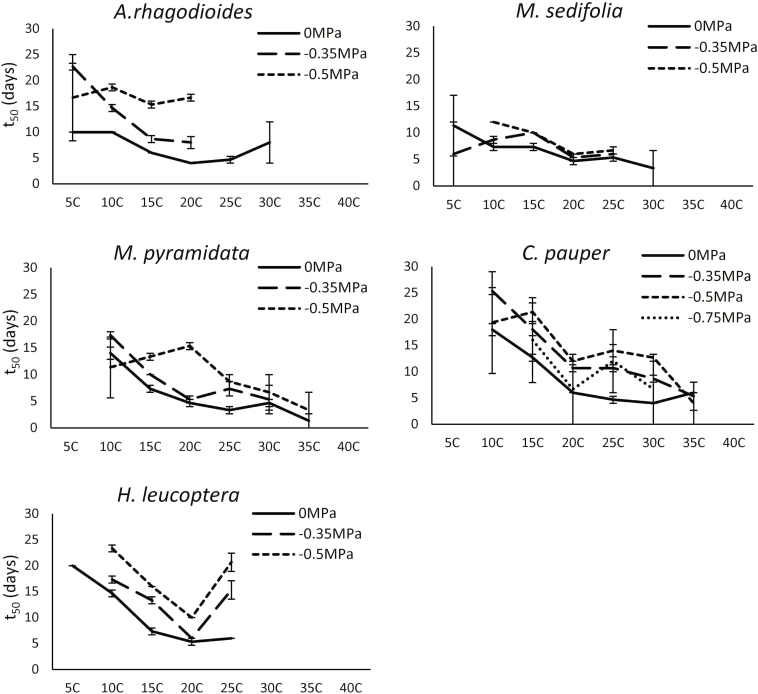
Time to 50 % germination (mean ± standard error) of *A. rhagodioides*, *M. sedifolia*, *M. pyramidata*, *C. pauper* and *H. leucoptera* seeds incubated under different water potential (0, −0.5 and −0.75 MPa) and temperature treatments (5–40 °C).

For most species in this study, germination speed increased with increasing temperatures, and decreased with decreasing water potentials. The exception to this pattern was *H. leucoptera*, which showed germination speed increasing with temperature, until a peak in germination speed at 20 °C that was followed by a rapid decline in germination speed as temperatures continue to rise (Fig. 4). Time to 50 % germination (*t*_50_) values for *H. leucoptera* at −0.5 MPa were twice that at saturated conditions, exhibiting the greatest delays due to water limitation of the five species. The two *Maireana* shrubs were the fastest germinating species, with *t*_50_ values less affected by reduced water potential than observed in all other species. Water stress generally increased *t*_50_ values, and variation in *t*_50_, and decreased maximum temperatures for germination. Delayed germination in response to reduced water potential was observed only at water potentials ≥ 0.35 MPa, and in conditions wetter than this, germination speed remained consistently high for all species.

### Cardinal temperatures and base water potentials for seed germination

**Figure 5.**
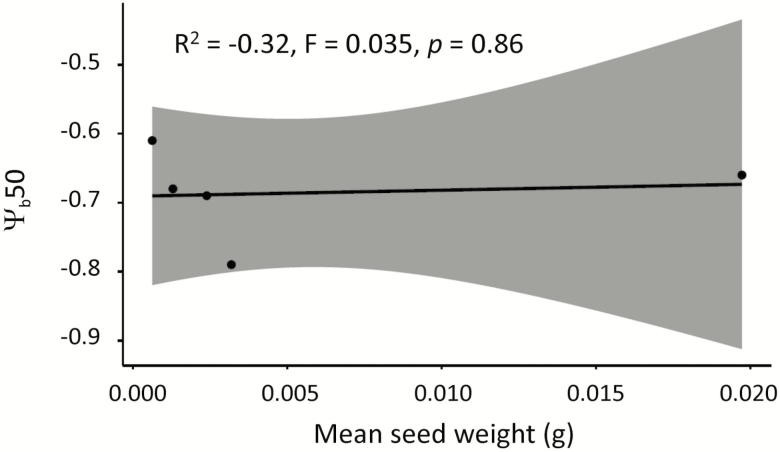
Linear model showing no relationship between mean seed weight (g) and base water potential to germination to 50 % (Ψ _b_50).

For all species, base water potentials for germination revealed low germination when conditions are dry. The base water potential for 50 % germination ranged between −0.61 and −0.79 MPa; hence, the driest condition at which seeds could germinate did not vary greatly between species. At temperatures above 20 °C, *M. pyramidata* and *C. pauper* were able to germinate in the driest conditions, with the lowest base water potential for germination. All species had low germination proportions (≤10 %) under low water potentials (−1.0 MPa) and no germination was observed at the lowest water potential tested, −1.5 MPa. There was no relationship between base water potentials for germination and seed mass (*R*^2^ = −0.32, *F* = 0.035, *P* = 0.86; Fig. 5). Average seed weight for *H. leucoptera* was 0.02 g, and seed weights of all other species are presented in Duncan *et al.* (2019).

Cardinal temperatures for seed germination revealed all species, except *C. pauper* and *M. pyramidata*, had low base temperatures for germination (*T*_b_ ≤ 5 °C; Table 1; **see****Supporting Information—Fig. S1**). Optimum temperatures for germination ranged between 15 and 31 °C, and were highest for *M. pyramidata* and *C. pauper*. Maximum temperatures for germination were greatly reduced with water limitation in *A. rhagodioides*. Species were able to germinate in dryer conditions at 15 or 20 °C, whereas seeds required more moisture to germinate at temperatures above 25 °C. All species showed higher germination proportions at optimal and suboptimal temperatures, than at maximum temperatures for germination. At supra-optimal temperatures seeds were more sensitive to water stress, with germination proportions declining more rapidly in drier conditions at ceiling temperatures for germination.

**Table 1.** Minimum (*T*_b_), optimum (*T*_opt_) and maximum (*T*_c_) temperatures for 50 % germination at three water potentials. Results show cardinal temperature estimates (°C) from segmented models. The adjusted *R*^2^ of each segmented model is shown, as well as the standard error of *T*_opt_.

Species	Segmented model adjusted *R*^2^	Cardinal temperatures (°C)		
		*T*_b_	*T*_opt_	*T*_c_
*A. rhagodioides*				
0 MPa	0.93	−0.7	22.6 ± 1.1	34.7
−0.35 MPa	0.99	−0.8	20.0 ± 0.3	25.0
−0.5 MPa	0.99	−8.8	17.7 ± 0.4	25.0
*M. sedifolia*				
0 MPa	0.91	−4.1	25.0 ± 0.9	30.0
−0.35 MPa	0.77	1.5	24.6 ± 1.2	30.0
−0.5 MPa	0.89	2.7	24.1 ± 0.8	30.0
*M. pyramidata*				
0 MPa	0.64	6.5	15.0 ± 3.8	34.7
−0.35 MPa	0.92	5.7	22.4 ± 1.0	30.0
−0.5 MPa	0.66	3.4	24.8 ± 2.9	38.9
*C. pauper*				
0 MPa	0.97	7.2	31.4 ± 0.8	40.0
−0.35 MPa	0.94	2.9	32.8 ± 0.8	40.0
−0.5 MPa	0.90	3.4	27.0 ± 1.4	39.7
*H. leucoptera*				
0 MPa	0.92	2.7	22.7 ± 0.9	30.0
−0.35 MPa	0.84	5.3	20.0 ± 1.7	29.7
−0.5 MPa	0.96	4.8	20.1 ± 1.1	29.9

Overall, the hydrotime model was a good predictor of germination behaviour under water stress **[see****Supporting Information—Fig. S2****]**, with the majority of *R*^2^ values exceeding 0.8 (Table 2). The hydrotime model often described germination behaviour at minimum and maximum temperatures better than it did at optimum temperatures. The model often failed to predict the consistently high germination of seeds at water potentials between 0 and −0.5 MPa. Generally the hydrotime model predicted lower base water potentials for germination than observed by the data.

**Table 2.** Hydrotime required to 50 % seed germination shows seeds accumulate more hydrotime under cooler conditions. θ _H_ = hydrotime (MPa h^−1^) to germination to 50 %; *R*^2^ = determination coefficient; Ψ _b_50 = base water potential to germination to 50 %; σ = standard deviation of Ψ _b_50 (MPa).

Species	Hydrotime parameter	5 °C	10 °C	15 °C	20 °C	25 °C	30 °C	35 °C
*A. rhagodioides*	θ _H_	170	260	95	49	41	21	52
	*R* ^2^	0.83	0.91	0.90	0.91	0.94	0.95	0.82
	Ψ _b_50	−0.66	−1.11	−0.79	−0.61	−0.47	−0.07	0.19
	σ	0.24	0.29	0.22	0.27	0.33	0.30	0.50
*M. sedifolia*	θ _H_	405	155	165	105	110	23	No germination
	*R* ^2^	0.84	0.81	0.86	0.67	0.76	0.91	
	Ψ _b_50	−0.81	−1.03	−1.19	−0.97	−1.32	−0.06	
	σ	1.14	0.27	0.34	0.35	0.61	0.25	
*M. pyramidata*	θ _H_	No germination	285	145	53	46	38	58
	*R* ^2^		0.89	0.92	0.72	0.85	0.86	0.80
	Ψ _b_50		−0.98	−0.89	−0.70	−0.68	−0.47	−0.36
	σ		0.57	0.41	0.23	0.32	0.55	0.79
*C. pauper*	θ _H_	No germination	465	365	105	55	45	50
	*R* ^2^		0.91	0.93	0.86	0.62	0.88	0.87
	Ψ _b_50		−1.14	−1.29	−0.83	−0.63	−0.62	−0.49
	σ		0.27	0.30	0.26	0.31	0.36	0.52
*H. leucoptera*	θ _H_	285	580	110	74	77	77	No germination
	*R* ^2^	0.90	0.83	0.64	0.85	0.86	0.87	
	Ψ _b_50	−0.58	−1.69	−0.78	−0.83	−0.62	−0.12	
	σ	0.15	0.36	0.22	0.15	0.18	0.22	

## Discussion

This study demonstrates the importance of the interaction between temperature and water availability in the germination responses of arid plant species. We predicted high moisture thresholds for germination as the dominant trait among the species in our study, which would prevent seedlings emerging during dry conditions. Indeed, for three species (*A. rhagodioides*, *M. sedifolia* and *H. leucoptera*) germination proportion and the thermal range for germination was drastically reduced when water potential was <−0.5 MPa (Fig. 2). Germination of these species is likely limited to large and rare rainfall events. Conversely, some tree species from semi-arid regions of eastern Australia, *Eucalyptus cambadgeana* and *Acacia harpophylla* (Arnold *et al.* 2014a, b), and *Banksia* species from western Australia (Cochrane *et al.* 2014) are remarkably tolerant to water stress and germinate at water potentials as low as −1.5 MPa. However, these species occur in regions with seasonal rainfall regimes, where the chance of follow-up rainfall is higher and the risks associated with germinating from small rainfall events are reduced. *Casuarina cristata* was considered water-stress-sensitive and showed no germination at water potentials below −0.75 MPa (Arnold *et al.* 2014b), similar to *C. pauper* and other species in this study. Many other arid species can germinate at remarkably lower water potentials than species in this study (Dürr *et al.* 2015; Hu *et al.* 2015; Shaygan *et al.* 2017), making them greater competitors when water is limited. Indeed, the hydrotime model predicted lower base water potentials for germination than actually observed. Hence, species in this study are generally considered water-stress-sensitive, and avoidance of dry and hot conditions is a key trait enabling them to persist in an environment where rainfall events are usually small and unpredictable.

### Germination speed reduced by water limitation

Rapid germination was observed in all species in this study at saturated conditions, and is an important advantage for arid species, because it enables them to capitalize upon the shorter pulses of water availability (Chesson *et al.* 2004). Germination rate for *A. rhagodioides* and *H. leucoptera* was greatly reduced by decreasing water potentials, taking twice as long to germinate at −0.5 MPa than at saturated conditions of 0 MPa. Slowed germination rate means seeds are exposed to a greater risk of seed death from desiccation; thus, delayed germination has strong fitness consequences (Donohue 2005; Hoyle *et al.* 2015). However, significant delays in germination in response to water limitation may be beneficial if seeds can survive extreme conditions for extended periods of time, assuming they are not lost to predation (DeFalco *et al.* 2012). The risk-avoidance strategy is particularly beneficial to species with high seed longevity, including *H. leucoptera*, as seeds may remain in the canopy, or soil, until a large rainfall event occurs. Reduced germination proportion and speed under severely water-limited conditions observed in our study are consistent with previous studies (Joel and Oscar 2001; Van den Berg and Zeng 2006). This cautionary approach to germination due to water limitation is considered a special survival strategy used by arid species to reduce seedling mortality after low rainfall events (Zeng *et al.* 2010).

Rapid germination is an important strategy for arid seeds and small seeds generally germinate faster than heavy seeds (Gomaa and Picó 2011). However, larger seeded species are often found in dry environments (Baker 1972) and produce seedlings with greater survival and establishment rates in dry conditions (Leishman and Westoby 1994; Moles and Westoby 2004). This suggests that larger seeds are more drought-tolerant, although few studies have explored the relationship between base temperatures and water potentials for germination and seed size. Certainly, some studies support this theory and show larger seeds have lower base water potentials for germination (Daws *et al.* 2008; Arène *et al.* 2017), which may enable them to exploit the advantages of increased survival rates at the seedling stage (Westoby *et al.* 1992; Baraloto *et al.* 2005). However, contrary to our prediction, there was no correlation between base water potential for germination and seed mass. Base water potentials for germination were similarly low for all species (Ψ _b_50 = −0.61 to −0.79 MPa), which suggests this is driven by the adaptive traits of arid seeds, rather than constraints in seed size, although our ability to ascertain the importance of the seed-size water-potential relationship is limited by the small number of species in this study.

### Thermal ranges narrowed by water limitation

Temperature is an important factor for regulating the timing of seed germination, and thermal ranges for germination vary among biomes. Most species had wide temperature thresholds for germination, probably because the probability of rainfall is equal across all seasons. Certainly, *T*_b_ values were low for all species (<8 °C), although *T*_c_ was lower for *M. sedifolia* and *H. leucoptera* than observed in most other native species (according to Dürr *et al.* 2015). This suggests that these two species have narrow thermal ranges for germination, which is driven by low *T*_c_ values. Water limitation altered temperature ranges for germination and generally induced two types of germination patterns; minimal changes to germination proportion and temperature thresholds for germination (*M. pyramidata* and *C. pauper*), or those with greatly narrowed temperature thresholds for germination and low germination proportions at low water availability (*A. rhagodioides*, *M. sedifolia* and *H. leucoptera*). Narrowed temperature ranges for germination due to water limitation further support our first prediction that seeds are cued to germinate only in high moisture conditions.

We also expected seeds to show higher germination proportions in cool temperatures, and this was true of all species in this study. This demonstrates a higher tolerance of water stress when temperatures are cooler, allowing seeds to capitalize on lower evaporation rates at winter conditions. For example, there was a sudden drop in germination above 30 °C for *A. rhagoidiodes*, *M. sedifolia* and *H. leucoptera*, which further supports selection for avoiding germination when evaporation rates are highest across summer. Negative temperature values for *T*_b_ reported here for *A. rhagodioides* and *M. sedifolia* are unlikely and exist because germination was high at 5 °C for these species and the linear model has failed to capture the rapid drop in germination that presumably occurs between 0 and 5 °C. Regardless, germination at the coldest temperature tested (5 °C) was high for *A. rhagodioides*, *M. sedifolia* and *H. leucoptera*, and supports our proposition that reduced germination at high temperatures is an important survival strategy for some arid species. We suggest that a preference for cooler temperatures and wet conditions are important characteristics of a risk-avoidance strategy because soil-moisture retention is greater in cold temperatures, thereby increasing the likelihood that seedlings emerge under optimal conditions for growth. However, soil temperature can be highly variable and influenced by soil surface humidity and moisture (Ashcroft and Gollan 2013); hence, we recommend further studies testing the germination responses of seeds in field conditions.

Bet-hedging refers to a seed’s ability to remain dormant or viable in the soil across seasons without committing to germination, and allows seeds to forego synchronous recruitment until conditions are suitable for plant establishment (Simons 2009; Gremer and Venable 2014). It is commonly observed in seeds from arid zones (Gremer *et al.* 2016; Fan *et al.* 2018; Lewandrowski *et al.* 2018) because, under low rainfall and high temperatures, germination is a high-risk event. These bet-hedging traits were observed in *H. leucoptera*; seeds have greater moisture requirements and lower germination proportions in hot conditions, and maintain high seed viability under physiological stress. *Hakea* is a serotinous species and has unusually high seed longevity (Duncan *et al.* 2019); hence, risk of germination failure can be avoided despite being a non-dormant species. Having a reservoir of seeds in the soil or canopy that germinate upon wetting, but can survive if moisture disappears prior to germination, may enhance recruitment opportunities under unpredictable rainfall events. Further research is required to understand seed persistence and the prevalence of bet-hedging in a greater suite of arid-zone species, and future studies should test these concepts *in situ*.

Serotiny enables species to control the timing of seed release with optimal conditions for seedling establishment, thus may be an important adaptation for arid-zone plants. Serotiny levels in *H. leucoptera* are not yet reported and, considering the interdecadal fire regimes at the study site, seed release is likely triggered by seasonal temperatures and/or humidity, rather than fire (Bradshaw *et al.* 2011). *Hakea* have several other drought adaptations that reduce their dependence on seed germination success, such as the ability to re-sprout from root suckers (El-ahmir *et al.* 2015), large seed sizes (Groom and Lamont 1997) and sclerophyllous, needle-shaped leaves (Barker *et al.* 1991) to prevent excess water loss. Compared to other species in this study, *H. leucoptera* has a tendency for reduced seed germination in dry conditions which, we suggest, contributes to its reservoir of drought-adapted traits (Lamont *et al.* 2016). Serotiny, seed longevity and high Ψ _b_50 values are all traits that delay seed germination and reduce seed mortality during drought.

### The risk-takers

The risk-taking germination strategy is typified by rapid germination across a wide range of conditions, and in response to lower rainfall events. In this study, two species (*C. pauper* and *M. pyramidata*) displayed these risk-taking characteristics. These species maintained wide thermal ranges for germination with moisture limitation and had higher germination proportions (55 and 38 %, respectively) in dry conditions (−0.75 MPa) than all other species. These species also had the highest maximum temperatures for germination at low water potentials and generally maintained rapid germination under water stress. Unlike *H. leucoptera*, both of these species had a large proportion of seeds die due to water limitation. This implies that *C. pauper* and *M. pyramidata* wager most of their seed reserves at each seeding event. However, for both species the risk-taking approach is advantageous because they seed reliably and frequently, and produce thousands of seeds at each seeding event (Wotton 1993; Callister 2004; Cunningham *et al.* 2011). The risk of population declines from extensive drought period is lessened by a steady supply of seeds, particularly for the long-lived tree species *C. pauper* that may have hundreds of seeding events throughout its lifetime. It has been suggested that higher functional plant species, such as perennial tree and shrub species, require larger rainfall events for establishment (Noy-Meir 1973; Schwinning and Sala 2004). These dynamics were not observed in this study as the slow-growing tree species, *C. pauper*, had similar moisture requirements for germination as *M. pyramidata*. Wide thermal and moisture envelopes for germination may explain the broader distribution in *C. pauper* and *M. pyramidata*. These two species also appear as the most dominant species of the region; hence, a risk-taking strategy (assuming consistently high seed supplies are maintained) may be the more successful survival strategy in arid zones with unpredictable rainfall events.

The importance of water thresholds as a survival strategy appears to be linked to other reproductive adaptations and trade-offs of species in this study. For example, *C. pauper* and *M. pyramidata* produce seed more reliably and frequently than other species in this study, and high seed production could mediate the impact and risks associated with lower Ψ _b_50 values for germination. Comparatively, flowering occurs very sparingly and irregularly for *M. sedifolia* (Wotton 1993), *H. leucoptera* (Barker *et al.* 1991) and *A. rhagodioides*; hence, their lower base water potentials and lower base temperatures for germination would be beneficial traits to allow more conservative use of limited seed production. To further mediate the effect of infrequent seeding events, *A. rhagodioides* has physiologically dormant seeds and *Hakea* have serotinous seeds that are generally long-lived, and have higher Ψ _b_50 values under increasing temperatures. Based on these results, we suggest that species that frequently and reliably produce seed can afford riskier germination strategies, such as germinating at lower water potentials, as the consequences of failure to establish are less dire than for species that do not reliably produce seed. Conversely, species with unreliable seed production avoid risk by limiting germination to wet conditions. We recommend further studies for a greater suite of arid species to test this relationship.

### Implications for restoration

As this study demonstrates, seed germination in arid zones is limited by high temperatures and low moisture availability, and these factors have been linked to poor recruitment outcomes from restoration efforts (García-Fayos *et al.* 2000; Chesson *et al.* 2004). However, water limitation impacts species in different ways and, for those that have high Ψ _b_50 values and avoid germination during dry conditions, restoration from seeding efforts alone remains challenging when rainfall is unpredictable. Re-establishment of species that avoid germinating in hot and dry conditions, such as *A. rhagodioides*, *M. sedifolia* and particularly for *H. leucoptera*, should be managed as water-stress-sensitive species with episodic recruitment. These species likely germinate following large rainfall events, which are rare in arid zones. As predicted by population models, plants with episodic recruitment require only 1.6 to 3.7 large recruitment events per century to sustain their population (Wiegand *et al.* 2004). For such species, further studies are required to investigate alternative intervention measures to support restoration (e.g. irrigation), and to understand the influence of climate change on the recruitment of water-stress-sensitive species in arid zones. Large-scale restoration methods usually involve a once-off application of seed (Corbett 1999), typically during autumn, when soil temperatures are still relatively warm and evaporation rates are lower. Our results suggest that cooler temperatures may enhance recruitment opportunities for drought-avoiding species in arid regions. Germination times may be marginally lengthened by cool temperatures but evaporation rates are lower, thus ensuring higher water retention in the soil profile beneficial for germination. Perennial species in this study exhibit varying responses to low moisture availability, suggesting that a ‘one for all’ approach may not be suitable for the restoration of all arid-zone species. Our results have demonstrated the physiological thresholds for germination of our study species, as determined in laboratory conditions. Further research should look to test these ideas in the field, where temperature, moisture and other environmental filters can rapidly fluctuate, impacting the germination responses of seeds.

## Data

The original data presented in figures and tables are available online at the TRY Plant Traits Database (https://www.try-db.org).

## Supporting Information

The following additional information is available in the online version of this article—

**Figure S1.** Rate of germination (to 50 %) at three water potentials (0, −0.35 and −0.5 MPa) and eight temperature treatments. Cardinal temperatures are estimated from the fitted segmented model; the lower and upper intercepts with the *x*-axis estimates *T*_b_ and *T*_c_, respectively, whereas the break in the segmented model estimates *T*_opt_.

**Figure S2.** Germination time courses across nine water potentials, with original germination data (dots) and hydrotime model predictions (lines) at three selected temperatures for each species (10, 20 and 30 °C for *A. rhagodioides*, *M. sedifolia* and *H. leucoptera*, and 15, 25 and 35 °C for *M. pyramidata* and *C. pauper*).

## Contributions by the Authors

C.D. developed the theoretical foundations and methods, conducted all experiments and took lead in analysing the data and writing the manuscript. N.S. and W.L. verified the methods and supervised the interpretation of results. All authors provided critical feedback and helped frame the research, analysis and manuscript.

## Sources of Funding

This project was funded by Tronox Holdings plc, as part of a PhD project aimed at understanding potential causes of high recruitment failures from seeding efforts.

## Conflict of Interest

None declared.

## Supplementary Material

plz066_suppl_Supplementary_Figures

plz066_suppl_Figure_Legends
