# Supplementary material for: The risk-takers and -avoiders: germination sensitivity to water stress in an arid zone with unpredictable rainfall
Source: AoB Plants. 2019 Oct 10;11(6):plz066. doi: 10.1093/aobpla/plz066 (PMC6863470; doi:10.1093/aobpla/plz066)
Supplement: plz066_suppl_Figure_Legends [file plz066_suppl_figure_legends.docx]

**Supporting Information**

The original data presented in figures and tables is available online at the TRY Plant Traits Database (<https://www.try-db.org>).

The following supporting information is available in the online version of this article:

**Figure S1:** Rate of germination (to 50%) at three water potentials (0, - 0.35 and - 0.5MPa) and eight temperature treatments. Cardinal temperatures are estimated from the fitted segmented model; the lower and upper intercepts with the x-axis estimates T_b_ and T_c_, respectively, whereas the break in the segmented model estimates T_opt_.

**Figure S2:** Germination time courses across nine water potentials, with original germination data (dots) and hydrotime model predictions (lines) at three selected temperatures for each species (10°C, 20°C and 30°C for *A. rhagodioides*, *M. sedifolia* and *H. leucoptera*, and 15°C, 25°C and 35°C for *M. pyramidata* and *C. pauper*).
